# Neuro-anaesthesia training in Pakistan: Evaluating the need for a dedicated fellowship program

**DOI:** 10.12669/pjms.41.5.10361

**Published:** 2025-05

**Authors:** Faraz Shafiq, Haris Sheikh, Khalid Ahsan, Tanveer Baig, Mohsin Nazir, Saqib Bakhshi

**Affiliations:** 1 Faraz Shafiq Department of Anaesthesiology, The Aga Khan University, Karachi, Pakistan; 2 Haris Sheikh Department of Anaesthesiology, The Aga Khan University, Karachi, Pakistan; 3 Khalid Ahsan Department of Anaesthesiology, The Aga Khan University, Karachi, Pakistan; 4 Tanveer Baig Department of Anaesthesiology, The Aga Khan University, Karachi, Pakistan; 5 Mohsin Nazir Department of Anaesthesiology, The Aga Khan University, Karachi, Pakistan; 6 Saqib Bakhshi Department of Neurosurgery, The Aga Khan University, Karachi, Pakistan

**Keywords:** Fellowship, Neuroanesthesia, Pakistan, Survey

## Abstract

**Background & Objective:**

Anaesthesia for neurosurgery has advanced with the passage of time. However, nation-wide training standards linked to general residency has certain limitations. It’s important to have dedicated neuro-anaesthesia fellowship program, basis of which should be the three pillars of neuro-anaesthesiology. The objective of this survey was to assess the need of neuro-anaesthesia fellowship based on opinions of practicing anaesthesiologists and neurosurgeons of Pakistan.

**Methods:**

After getting ethical approval and consent, data related to this cross sectional survey was collected using online questionnaire at the Aga Khan University, Pakistan. The form was circulated through social media to individuals and relevant groups. The survey link remained active for a duration of one month, from November 3rd, 2023, to December 3rd, 2023.

**Results:**

Total 282 forms were received, out of which 63 were incomplete while 219 were analyzed. 80.2% respondents were anaesthesiologists and 19.8% neurosurgeons. A significant finding was utilization of non-neuro-anaesthesiologist based clinical care for majority of surgical cases. Gaps in training during residency years were also identified. Survey participants also agreed that neurosurgical case setup is complex, which mandates the need of specialized knowledge and skills. Overall, 87% of participants favored the need of neuro-anaesthesia fellowship training. In general, 45.2% anaesthesiologist wished to pursue a further fellowship in neuro-anaesthesia, and 65% believed that one-year duration to be adequate. Only 7.0% of physicians proposed that there is no need of additional fellowship training.

**Conclusion:**

The need of neuro-anaesthesia fellowship program is well established based on findings of this survey. This was linked to training gaps as a part of general residency curriculum. Dedicated fellowship program is required for improving patient safety and better outcomes.

## INTRODUCTION

Neuroanaesthesia is an evolving field that is becoming increasingly sophisticated, not just in terms of numbers involved but also in complexity regarding techniques and approaches used.^1^ Advancement in neurosciences is coming up with more complex scenarios, including functional and stereotactic surgical procedures. The impact of which would be on better neurological outcomes and enhancing quality of life.^2^ Expertise in such scenario is important, for careful patient selection, facilitation in intraoperative neurological monitoring, and prevention of related complications.^3^ Globally, neuro-anaesthesia training is tied to standard residency curriculum, mostly required two to three months of clinical rotation. This leaves residents with limited exposure and expertise.^4^ The introduction of subspeciality fellowship programs in anaesthesia has been popular and heading effectively for many academic platforms. The neuro-anaesthesia fellowship follows the same pattern and has now become a part of many renowned anaesthesia departments globally.^5^

**Figure:**
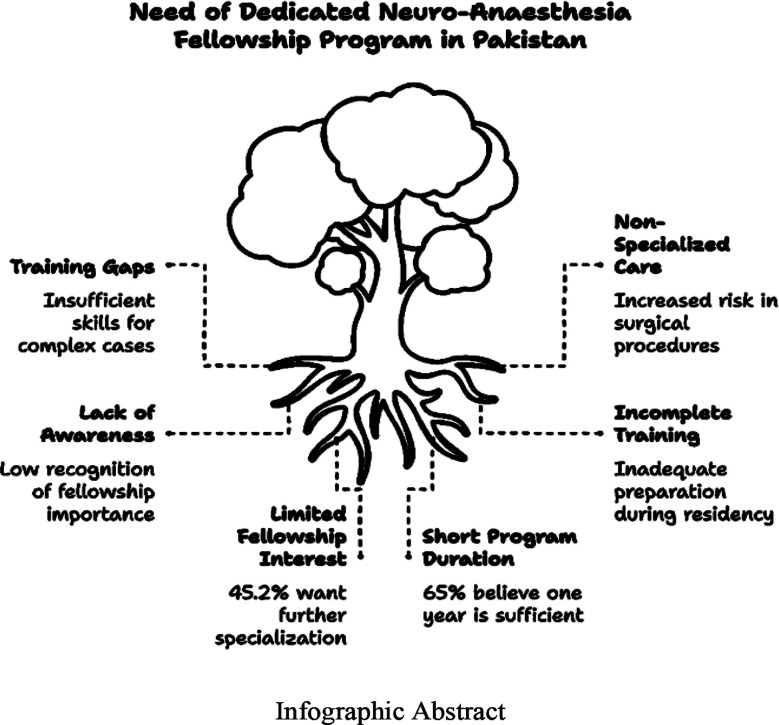
Infographic Abstract

In Pakistan, training standards are often compromised due to a lack of resources, even for basic anaesthesia training.^6^ Moreover, the disease burden is substantial with considerable amount of case load. This is evident by around seventy-two neurosurgical centers in Pakistan, with overall bed capacity of fifteen hundred patients.^7^ As mentioned earlier, significant proportion of patients now require operative management with addition of complex cases like awake craniotomy (AC) and deep brain stimulation (DBS).^8^ The situation raises concerns about patient safety and quality of care provided to this group of population. This requires adequate planning to enhance neuro-anaesthesia training program and addressing ongoing challenges. Trained and competent neuro-anaesthesia team can match ongoing needs and play better role in enhancing care of these patients. It emphasizes the foundational concept of “The Three pillars of Neuro-anesthesiology” proposed by Dr. George Mashour, which should serve as a guiding framework for developing a neuro-anaesthesia fellowship program.^9^ Foundation pillars include specialized training required for care of neurosurgical patients, understanding molecular basis, and improving patient related outcomes by limiting anaesthesia toxicity. This can only be achieved by having dedicated fellowship program.

The objective of this survey was to assess the need of neuro-anaesthesia fellowship program and related factors based on opinions from practicing anaesthesiologist and neurosurgeons of Pakistan.

## METHODS

The data collection for this survey was conducted using RedCap software, which provided a user-friendly platform for participants to submit their responses. The targeted population included anaesthesia and neurosurgery trainees, specialists, and consultants practicing in Pakistan.

### Ethical Approval:

Prior to the commencement of the survey, approval was obtained from The Aga Khan University ethics committee (Reference: 2023-9342-26810). Informed consent was also secured from all participating physicians, ensuring that they were fully aware of the survey’s purpose.

The survey link was disseminated online through WhatsApp accounts of individuals, relevant professional societies, and focused groups. Primary investigators utilized their personal contact lists to encourage participation, ensuring a broad reach within the target demographic. The expected time for completion of the survey was estimated to be between five to seven minutes. The survey link remained active for a duration of one month, from November 3rd, 2023, to December 3rd, 2023. During this period, participants received two reminders to encourage completion of the survey, thereby enhancing response rates. The data collection form included several key components, which are detailed below. A supplementary file containing the full survey form is attached for reference.

1. Consent
2. ***Demographic Variables:*** Collection of demographic information relevant to the participants.
3. ***Assessment of Clinical Services:*** Evaluation of the current clinical services in relation to Neuroanaesthesia.
4. ***Neuro-anaesthesia Training During Residency:*** Assessment of the training received by participants initially in Neuro-anaesthesia.
5. ***Assessment:*** Regarding the Need for a Neuroanaesthesia Fellowship Program in Pakistan: Gathering opinions on the necessity of establishing a dedicated fellowship program in Neuro-anaesthesia.

The data was entered and analysed through Statistical Package for Social Science (SPSS) version 19 (Chicago, Illinois, USA). A descriptive analysis was conducted, focusing on the frequency and proportions of responses within the various sections: demographics, clinical services, neuro-anaesthesia training during residency, and the demand for the neuro-anaesthesia fellowship program.

## RESULTS

In total, the survey link was distributed to 425 participants, out of them 282 responded. While 63 forms were incomplete. Estimated response rate of this cross-sectional survey was 66% and 219 responses were included for final analysis. Amongst these, 80.2% were anaesthesiologists and 19.8% were neurosurgeons. The regional distribution, professional affiliation, academic rank, and experience of participants is mentioned in (Table-I). About 83.1% respondents have their specialized training from College of Physicians and Surgeons Pakistan. Regional representation revealed most of respondents were from (56%) Sindh province.

**Table-I:** Demographic data of participants.

	Anaesthesiologist (N=177)	Neurosurgeon (N=42)	Overall (N=219)
*Years of experience*			
10 - 15 years	15 (8.5%)	1 (2.4%)	16 (7.3%)
5 - 10 years	29 (16.4%)	17 (40.5%)	46 (21.0%)
Greater than 15 years	65 (36.7%)	4 (9.5%)	69 (31.5%)
Less than 5	60 (33.9%)	18 (42.9%)	78 (35.6%)
*Institution*			
Private and non-teaching	5 (2.8%)	4 (9.5%)	9 (4.1%)
Private and Teaching	106 (59.9%)	23 (54.8%)	129 (58.9%)
Public Sector and Non-teaching	5 (2.8%)	3 (7.1%)	8 (3.7%)
Public Sector and Teaching	60 (33.9%)	12 (28.6%)	72 (32.9%)
*Province*			
Baluchistan	1 (0.6%)	1 (2.4%)	2 (0.9%)
KPK	19 (10.7%)	15 (35.7%)	34 (15.5%)
Punjab	56 (31.6%)	2 (4.8%)	58 (26.5%)
Sindh	101 (57.1%)	23 (54.8%)	124 (56.6%)
AJK	0 (0%)	1 (2.4%)	1 (0.5%)
*Grade*			
Medical Officer	7 (4.0%)	2 (4.8%)	9 (4.1%)
Registrar	5 (2.8%)	5 (11.9%)	10 (4.6%)
Resident	57 (32.2%)	9 (21.4%)	66 (30.1%)
Senior Instructor/AP/Associate/Professor	90 (50.8%)	19 (45.2%)	109 (49.8%)
Specialist/Instructor	18 (10.2%)	7 (16.7%)	25 (11.4%)
*Highest Qualification (Diploma/Degree)*			
Diplomat American Board	2 (1.1%)	1 (2.4%)	3 (1.4%)
FCPS	149 (84.2%)	33 (78.6%)	182 (83.1%)
FRCA	3 (1.7%)	0 (0%)	3 (1.4%)
MCPS	8 (4.5%)	0 (0%)	8 (3.7%)
FRCS	0 (0%)	2 (4.8%)	2 (0.9%)

**Fig.1:**
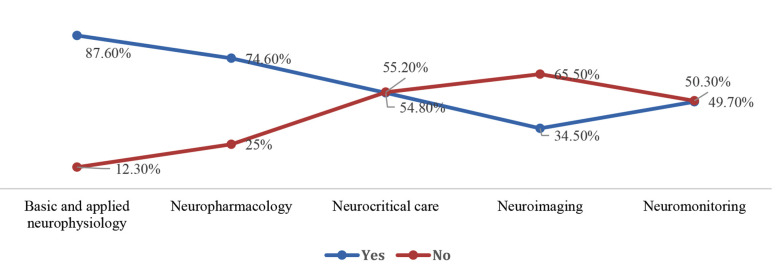
Foundations of Neuroanaesthesia covered during general training.

**Fig.2:**
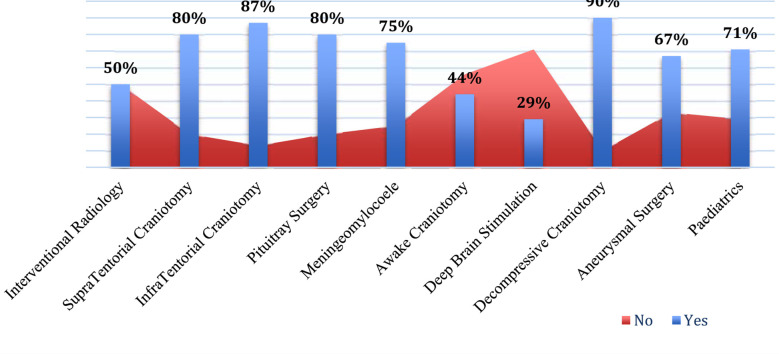
Experience of Aanesthesiologists about complex neurosurgical cases.

**Fig.3:**
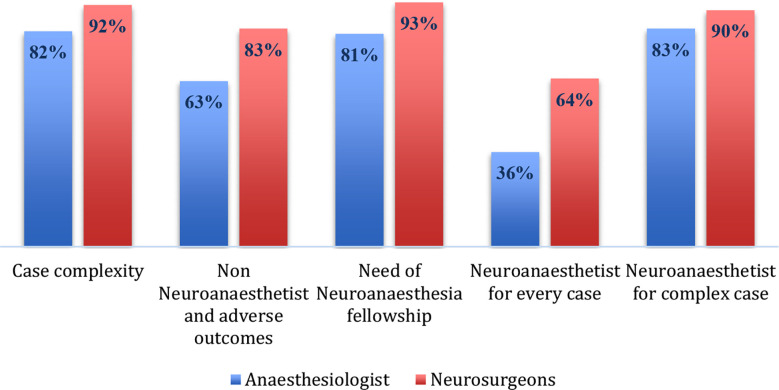
Comparison of agreement regarding case complexity, need of neuro-anaesthetist and fellowship program in neuro-anaesthesia.

Majority of them (60%) had working experience of more than five years. While many of them (47.5%) were from consultant grade and had affiliation with teaching hospitals (71%). The facilities for diagnostic radiology, operative management, and ICU existed in most of the clinical setups (Table-II). However, only 43% of physicians reported the provision of dedicated neuro-anaesthesiologist in operative list. Amongst them, only 8% had fellowship or any additional training (16%) in neuro-anaesthesia. Majority of anaesthesia respondents reported that aspects related to neurophysiology and pharmacology had been covered adequately during initial residency training (87.6:74.6%). More importantly, gaps were identified in curriculum related to neurocritical care, imaging and neuroanatomy as a part of primary training (Fig.1). The responses were 54.8%, 34.5% and 49.7% respectively. Almost 48.5% anaesthesiologist were satisfied about number of neurosurgical cases they have experienced during residency. Lack of exposure was also identified for cases like interventional radiology, DBS, AC, and aneurysmal surgery respectively (Fig.2). Both, anaesthesiologists, and neurosurgeons were agreed (85.2%:92.8%) that neurosurgical case setup is complex in terms of patient positioning, monitoring and neuroprotection. Hence, majority of them favoured the need of neuro-anaesthetist for better outcomes. The need of fellowship program revealed clear agreement between (80.8%) anaesthesiologist and (92.9%) neurosurgeons (Fig.3). Amongst anaesthesiologists, 45.2% wished to pursue a further fellowship in neuro-anaesthesia, and 65% believed one year should be an adequate duration for that. Only, 7.0% physicians proposed that there is no need of any additional fellowship training. They mentioned reasons pertaining to no added benefit (61%), financial needs (46%), delay in becoming independent consultant (2.8%) and few have proposed modification in existing curriculum (1.2%) (Table-III).

**Table-II:** Status of available Neuro-services.

Available Services	Percentages %
Dedicated Neuro-surgery Operative List	86%
Emergency Neurosurgery Care	87%
Neuro-intensive Care	51%
Diagnostic Radiology	92%
Interventional Radiology	63%
Dedicated Neuro-anaesthetist	43%
Fellowship in Neuro-anaesthesia	8%
Second Fellowship in Anaesthesia	47%
Additional training in Neuroanaesthesia	16%

**Table-III:** Reasons for not favoring fellowship program.

Reasons	Frequency (%) N=15
Financial needs	6 (40%)
Extra Burden	7(47%)
Prolong Duration	6(40%)
Better opportunities	5(33%)
Delay in becoming consultant	5(33%)
No added benefit	8(53%)

## DISCUSSION

The findings of survey highlight the significant training gaps in Neuroanaesthesia with in Pakistan. It emphasizes the necessity for specialized training programs to enhance the skills and knowledge of healthcare professionals in this critical field. The results indicate a strong consensus among respondents regarding the establishment of a Neuroanaesthesia fellowship program, which is essential for improving patient outcomes in neurosurgery. The results clearly mentioned the overall training gaps, and the need for specialized training in Neuroanaesthesia in Pakistan. Considering the structure of the national training program and the available resources, advanced healthcare setups in the country should have a provision for such program. This is important to build the necessary concepts required to improve the outcomes of neurosurgical patients.^10^ Being a neuro-anaesthesiologist requires a command of the basic understanding of pathophysiological processes and the necessary skills to address associated perioperative challenges.^11^ The majority of respondents also favored the establishment of a Neuroanaesthesia fellowship in Pakistan, underscoring the demand for structured training that can equip practitioners with the expertise needed to excel in this specialized filed of anaesthesia. Most of the participants were qualified, had adequate experience, and were working at a consultant grade, which enhances the validity of the survey. This also reflects their interest in progression in academics and research, as shown by similar survey already conducted elsewhere.^12^ As revealed, the primary residency training currently does not cover the necessary skills and experience required for neuro-anaesthesia.^4^ This, combined with already compromised postgraduate training, contributes to deficits in necessary skills and achieved competencies.^13^ Approximately 38% of anaesthetic physicians expressed dissatisfaction with the number of cases they encountered during residency training. Similarly, the majority of respondents were not satisfied with the technical aspects of neuro-anaesthesia covered during that period. Many felt inexperienced in anaesthetizing cases such as Aneurysmal Clipping, DBS, and providing care for procedures involving Interventional Radiology (IR). To meet the growing needs and demands, a competent anaesthesia team is required, with special interest, adequate training, good communication skills. Nonetheless, dedicated fellowship program will be the platform for capacity building and developing future leaders. As an ongoing engagement, the opportunity is important ultimately for staff retention and future planning.^14^ The survey results also mentioned available resources in terms of basic neurosurgery-related facilities. Most setups had provisions for dedicated operative lists and related diagnostics including postoperative intensive care facilities. However, it is imperative to understand that nationwide distribution of these facilities is not uniform. Very few centers are performing complex procedures. In light of these facts, it is essential for advanced teaching setups to promote the concept of fellowship programs. Most participants agreed that a fellowship program is a way to improve patient-related outcomes, as has already been experienced in other subspecialties.^15^ The results also validated the concept of “Three pillars of neuro-anaesthesiology” and reinforced the development of future programs based on this framework. Globally, attracting physicians for fellowship training programs is challenging. 16 The reasons behind are multifactorial, including financial concerns and a lack of interest or understanding that adequate learning has been achieved during residency years. Similar observations were made in this survey, with a few participants suggesting changes to the current residency curriculum. However, integrating the three-pillar approach into a constrained residency program is challenging. A minimum of one year is required to cover the core curriculum and learn the technicalities of complex neuro-anaesthesia cases which can only be achieved through a dedicated fellowship program.^17^ After completing residency training, majority of postgraduates wish to start their careers at the consultant level. Historically, remuneration and financial issues have been identified as concerns regarding retention and should be part of workforce planning.^18^ A dedicated fellowship program would facilitate international collaboration and networking, providing a significant opportunity for fellowship trainees from low- and middle-income countries (LMIC) like Pakistan.^19^ Furthermore, research and skill set requirements of the fellowship curriculum would be appealing components of proposing this fellowship program. We believe that future proposals for similar programs should emphasize the necessary skill set that fellows would acquire during their training to address financial aspects and other barriers associated with induction.

### Limitations:

There were certain limitations associated with the survey. Despite achieving a response rate of 66%, which fell short of expectations, the findings provide valuable insights into the topic at hand. Physicians were randomly selected based on a personal contact list, which likely resulted in an incomplete representation of the actual number of physicians practicing nationwide. Consequently, a significant portion of the responses originated from Sindh Province, potentially skewing the results. The exclusive use of WhatsApp as a distribution medium may have further limited outreach, as it may not encompass all demographics of physicians. To enhance the effectiveness of future surveys, a more pragmatic approach to distribution is recommended. Utilizing official channels or involving regional representatives could broaden the reach and ensure a more comprehensive representation of physicians across the country. In terms of study design, the analysis primarily relied on descriptive statistics, which introduces the potential for bias in interpreting the results. Despite these limitations, the insights gained from this survey is invaluable. More importantly, by knowing these limitations the results give insight to current status and further exploration of this very important topic in future.

## CONCLUSION

The findings of this report highlight the need for specialized neuro-anaesthesia fellowship training to enhance the safety and efficacy of neurosurgical care. By recognizing the limitations of general anaesthesia training and addressing the barriers to pursuing further education, we can work towards improving clinical outcomes for neurosurgical patients through dedicated neuro-anaesthesia programs.

### Author’s Contributions:

**FS:** Conceived the study, worked on the study design, helped with data collection, wrote and approved the final manuscript and responsible and accountable for the accuracy or integrity of the work.

**HS:** Involved in ethical approval process, designed the questionnaire, helped with data collection, performed the analysis and wrote the final results.

**KA** and **TB:** Helped with data collection, proof reading of final manuscript.

**MN:** Involved in approval process from research committee, data handling and proof reading.

**SB:** Involved in the dissemination of survey link to neurosurgical community and data collection from that cohort, proof reading of final manuscript.
